# Construction and Evaluation of Intelligent Medical Diagnosis Model Based on Integrated Deep Neural Network

**DOI:** 10.1155/2021/7171816

**Published:** 2021-11-25

**Authors:** Lina Ma, Tao Yang

**Affiliations:** ^1^Henan Medical College, Zhengzhou, Henan 451191, China; ^2^Henan University of Traditional Chinese Medicine, Zhengzhou, Henan 450000, China

## Abstract

In recent years, as human life expectancy increases, birth rate decreases and health management concerns; the traditional Healthcare imaging system, with its uneven Healthcare imaging resources, high Healthcare imaging costs, and diagnoses often relying on doctors' clinical experience and equipment level limitations, has affected people's demand for health, so there is a need for a more accurate, convenient, and affordable Healthcare imaging system that allows all people to enjoy fair and quality Healthcare imaging services. This paper discusses the construction and evaluation of an intelligent medical diagnostic model based on integrated deep neural networks, which not only provides a systematic diagnostic analysis of the various symptoms input by the inquirer but also has higher accuracy and efficiency compared with traditional medical diagnostic models. The construction of this model provides a theoretical basis for integrating deep neural networks applied to medical neighborhoods with big data algorithms.

## 1. Introduction

Currently, artificial neural network (ANN) is one of the research activities in the artificial intelligence neighborhood, which is a mathematical model of artificial intelligence similar to synaptic connections constructed by simulating the nervous system of a living organism, and this algorithm is like a brain composed of countless neurons, with the ability to perform large-scale operational processing, store information, and have good self-organizing learning ability, among other characteristics [1]. The emergence of neural networks has led to attempts to free computers from the mechanical execution of programs so that they can be used in a wide range of areas of life.

Deep neural network (DNN), as an enhanced version of neural networks, is a neural network with many hidden layers, which is also known as deep feed-forward networks (DFNs), and because neural networks are called perceptron, DNNs are also known as multilayer perceptron (MLP), which can also be referred to as a fully connected deep network because of the fully connected connections between its neurons [2]. It should be noted that DNN is not the name of a certain neural network, but a general term for a class of neural networks, which has a wide range of concepts and includes many categories, such as convolutional neural networks (CNNs) and recurrent neural networks (RNNs), which are often used in applications [3] and so on, and all fall within this category.

Deep neural networks are compared with neural networks by adding a hidden layer [4], it also has more than one neuron in the output layer, and it has been extended in the activation function to achieve the change from shallow neural networks to deep neural networks, solving the problem of small capacity of neural networks and most importantly overcoming such fundamental problems as linear indistinguishable heterogeneity, so that the model is better trained and optimized, strengthening the expressive power [5]. In the structure of a fully connected DNN, all neurons in the upper and lower layers, regardless of the number, are interconnected, which can be imagined in complex neural network models where the number of parameters can be so large that it can be overwhelming and even cause catastrophic problems [6]. For example, when an image with too large pixels is input, the weight of the training weights for one layer is large, a situation that not only leads to overfitting but also to the inability to find a global optimal solution and walking into a local optimal dilemma [7].

With the development of computers and biological advances in deep neural networks, theoretically enriched expansion of deep neural networks and relevant concepts in image processing were added to neural networks, namely, convolutional kernels, which act as intermediaries between images and neural networks, sharing content through convolutional kernels, rather than being directly connected as in fully connected DNNs. [8]. We refer to it as a convolutional neural network [9]. The same idea can be applied in neighborhoods such as computer language recognition and natural language processing.

Therefore, the research and applications of deep neural networks in medical data are becoming increasingly widespread in smart Healthcare imaging facilities and infrastructures and can be broadly classified into the following categories: disease prediction, disease diagnosis, disease healing assessment monitoring, new drug development, health management, and medical imaging [10].

In the traditional Healthcare imaging environment, it is often faced with imbalance in medical resources, difficulty in accessing medical care, tension between doctors and patients, and high workload of medical workers [11]. Especially in medical diagnosis, modern medicine expects to treat and control diseases through early detection and early treatment and a low-cost and efficient concept of early intervention [12], but in traditional medicine, medical diagnosis is often limited by the experience of doctors and medical conditions, resulting in a low level of accuracy.

However, based on integrated deep neural networks and making full use of medical big data and authoritative experts and medical literature [13], the construction of an artificially intelligent medical diagnosis model can make up for the deficiencies that may be brought about by experience and equipment when humans are doctors and achieve a truly interactive information-based intelligent medical platform [14], allowing the timeliness and accuracy of diagnosis to be greatly enhanced and improved. The intelligent medical diagnosis model based on integrated deep neural networks constructed in this paper can systematically evaluate and analyze the symptoms presented by patients and provide a theoretical basis for big data algorithms to prevent the occurrence of other diseases and further improve and explore the intelligent medical neighborhood.

## 2. The Related Works

Deep learning is not a new research neighborhood in recent years, as early as the 1940s, there has been the emergence of related concepts and theoretical studies [15], and its development has now broadly gone through three phases: the budding period, the development period, and the boom period [16]; the budding period occurred in the 1940s to the 1960s; we first found the prototype of deep learning in cybernetics. The first linear artificial neuron simulating human nerves, the McCulloch-Pitts (M-P) neuron, were proposed by Chandrasekhar and Suresh [17], and later Alarifi et al. [18] proposed the first artificial neural network model that could learn the perceptron model, but because of its limited capacity and linear structure, it neither can segment heterogeneous problems, nor can it find a good learning algorithm to train multilayer perceptron to change the current situation, which leads to the suspension of research. The development of deep learning was stalled again.

After the emergence of connectionism, deep learning absorbed this idea so that neural networks finally started to evolve again in the 1980s [19], this time centering on the simulation of the nervous system of living organisms and distributed representation and the latter being also the core idea of the now popular deep learning. This phase of MLP became popular in 1988 when Rumellhart [20] and others deduced the error back propagation (BP) algorithm, but with a period of applied research, researchers found that the BP algorithm was not a good solution to the shallow network performance, MLP did not achieve the desired training effect, and the second boom declined.

The third wave of development, which lasted until 2006, really started the reboom of deep learning. The renaissance was marked by the emergence of deep belief networks (DBNs), a neural network that is an unsupervised probabilistic production model proposed by Hinton [21] and other researchers to achieve its learning by means of a layer-by-layer constrained Boltzmann machine [22]. A deep neural network can be constituted by adding a target-dependent output layer to the trained deep confidence network. Deep neural networks are made easy to train and optimize because of the addition of DBNs, allowing one to further exploit the structural advantages in deep neural networks.

The convolutional neural network we apply in this paper is from the second wave of development, that is, from the 1980s to 1990s, and its origins were also influenced by the study of visual cortex in real life [23, 24]; although some progress has been made, the model of convolutional neural network still suffers from many limitations, and it was only after the backpropagation algorithm mentioned above was applied to that the CNN began to gradually move to various application neighborhoods.

Models based on deep neural network algorithms are also becoming better and better in various neighborhoods of smart Healthcare imaging, with increasing accuracy. For example, in disease prediction, Dr. Weng's team from the University of Nottingham's epidemiology [25] used clinical data from 378,256 patients from UK households to train a set of deep neural network models for assessing cardiovascular disease risk and compared them with other types of machine learning algorithms at the same time and found that the deep neural network algorithms performed the best with an AUC value of 77%. In medical diagnosis, Xiao et al. [26] from Shenzhen University based on deep neural networks for computer-aided diagnosis of cancer proposed that image classification techniques have performed well in medical diagnosis studies of cancer and show great potential to be applied to improve clinical diagnosis in the future.

In summary, the application of integrated deep neural networks is getting deeper and deeper with the development of science and technology and has great potential for development and large expansion in the intelligent medical neighborhood. In this paper, a brief development history and basic principles of deep neural networks are introduced. In addition, this study investigates a model applied to intelligent medical diagnosis based on the advantages of this algorithm and evaluates and analyzes it.

### 2.1. Construction of Imaging Facilities and Infrastructure Based on Integrated Depth Neural Network Algorithm

**Figure 1:**
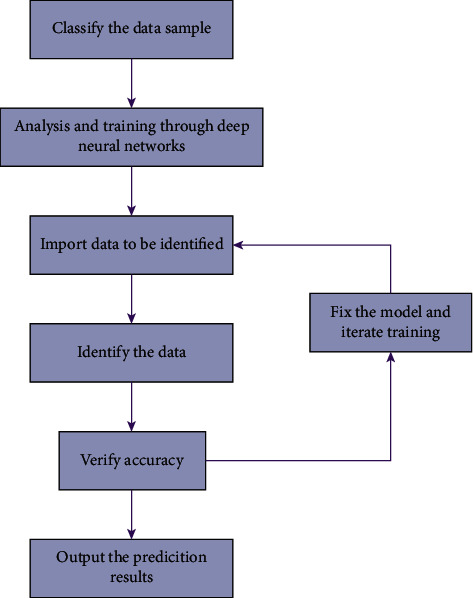
The constitution of deep neural network systems.

According to the practical needs, this paper will use the deep neural network model to model the data. The schematic diagram is shown in Figure 1.

As we can see in the figure, the basic steps include acquisition and preprocessing of data; training configuration through the designed network structure, i.e., finding the optimizer; putting the data into the model for training, with each round including three parts of forward calculation, loss function, and backward propagation in the loop call training; and finally, it is to save this trained model and call it to get the desired prediction when it is needed. Finally, the trained model is saved and called to get the desired result when it is needed.

### 2.2. Deep Neural Networks

The basic information processing structure of a neural network is the neuron, and the prototype is a biological neuron whose transmission is forward propagation, as shown in the following equation:

(1) $$\begin{array}{c} y=f\left(\sum_{k=1}^{n-1}w_{k}x_{k}-\theta \right). \end{array}$$

The deep neural network is an extension of the model of perceptron, the hidden layer is added, and the output layer can have more than one neuron, and the activation function is extended to further enhance the expressive power of the neural network.

**Figure 2:**
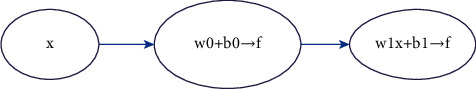
Operation diagram of deep neural networks.

DNN is a feed-forward network, which is composed of three parts: an input layer, an output layer, and several hidden layers.  Figure 2 is the schematic diagram of DNN operation.

**Figure 3:**
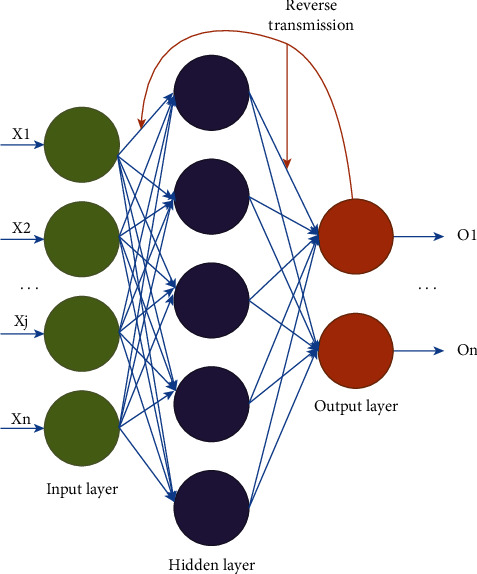
Operation diagram of back propagation.

After the input signal, the signal is propagated through the various layers to obtain the final output result. In the neural network, it mainly contains the forward propagation of the signal as in equation (1), and there is the error back propagation formula that will be used in the training of the model below. The back propagation schematic is shown in Figure 3.

In this process, the network first needs to be initialized and set up with parameters for each neuron {*w*, *a*, *θ*}.

A random initial value between (0,1) is assigned, and then the training data are input, and the forward propagation of the signal is performed to obtain a forward output *o*. Each training set of *x*, corresponding to the output layer unit *k*, has a target output *t* where the error is calculated as shown in the following equation:

(2) $$\begin{array}{c} \delta_{k}\;⟵\;o_{k}\left(1-o_{k}\right)\left(t_{k}-o_{k}\right). \end{array}$$

The error is passed backwards to the hidden layer *h*, as shown in the following equation:

(3) $$\begin{array}{c} \delta_{h}\;⟵\;o_{h}\left(1-o_{h}\right)\sum_{k\in outputs}w_{kh}\delta_{k}. \end{array}$$

The weights are adjusted using the propagated errors, as shown in the following equation:

(4) $$\begin{array}{c} w_{ji}\;⟵\;w_{ji}+△w_{ji}=w_{ji}+\eta \delta_{j}x_{ji}. \end{array}$$

The whole process of neural network training is actually the process of shrinking the loss.

(5) $$\begin{array}{c} \text{Loss}=\sum_{i=1}^{n}\left(y_{i}-\left(wx_{i}+b\right)\right). \end{array}$$

In order to minimize loss, we need to solve for the optimal linear relational coefficient matrix *w* and the bias vector *b*. We use the gradient descent solver to obtain *w* and *b* as follows:

(6) $$\begin{array}{c} \left(w_{0},y_{0}\right), \end{array}$$

(7) $$\begin{array}{c} b_{1}=b_{0}-\alpha \frac{\partial \text{Loss}\left(w,b\right)}{\partial b}. \end{array}$$

Finally, the DNN output is then performed by an activation function, which is commonly used in the same way as the convolutional neural network explained below.

### 2.3. Convolutional Neural Network

DNNs are prone to fall into overfitting and poor network generalization due to the excessive number of parameters and the limitation of the number of network layers, so it is necessary to introduce other operations into the network and deepen the number of layers in the network at the same time.

**Figure 4:**
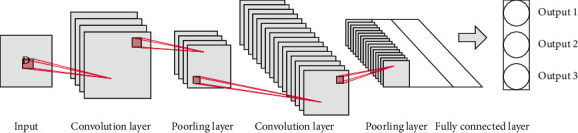
Convolution neural network architecture.

Convolutional neural networks have the advantage of being different from other deep neural networks in which they have a strong representational learning capability and can perform translation-invariant classification of the input information according to its hierarchical structure. The experimental ideas are local connectivity, weight sharing, and downsampling, and they contain multiple hidden layers, while computational operations such as convolution and pooling are introduced in the hidden layers to reduce the dimensionality of the image, reduce the parameters of the network, and enhance the neural network's training efficiency. The hidden layers composed of convolutional and pooling units are called convolutional and pooling layers, respectively. Convolutional neural networks are often used in the fields of computer vision, natural language, and language processing.  Figure 4 shows the architecture of a convolutional neural network.

Convolution layer (Conv): convolution is a computational method in the field of mathematics, including continuous convolution and discrete convolution, as shown in the following equations:

(8) $$\begin{array}{c} y\left(t\right)=\int {-\infty }_{\infty }x\left(p\right)h\left(t-p\right)=x\left(t\right)c⊗h\left(t\right), \end{array}$$

(9) $$\begin{array}{c} y\left(n\right)=\sum_{-\infty }^{\infty }X\left(i\right)h\left(n-i\right)=x\left(n\right)⊗h\left(n\right). \end{array}$$

In a convolutional neural network, the convolutional operation is implemented with the help of a convolutional kernel, as shown in the following equation:

(10) $$\begin{array}{c} x_{j}^{l}=f\left(\sum_{i\in M}X_{i}^{l-1}*K_{i}^{l-1}+b_{j}^{l}\right). \end{array}$$

The convolution and pooling operations described above achieve linear processing of convolutional neural networks, but since data such as images may be nonlinear and indistinguishable, nonlinear variations are introduced through the activation function in the operation of the network as a way to enhance the generalization capability of the network. There are three activation functions frequently used in deep neural networks, which are the following equations:

(11) $$\begin{array}{c} \text{sigmoid}\left(x\right)=\frac{1}{1+e^{-x}}, \end{array}$$

(12) $$\begin{array}{c} \tanh\left(x\right)=\frac{e^{x}-e^{-x}}{e^{x}+e^{-x}}, \end{array}$$

(13) $$\begin{array}{c} \text{relu}\left(x\right)=\max\left(0,x\right). \end{array}$$

Among them, the ReLU function is closest to the stimulus response of the brain receiving the signal source in biology and has a more desirable signaling effect in convolutional neural networks. For the error back propagation of convolutional neural networks, the error function needs to be defined. In order to reduce the overfitting phenomenon in training, the error function used in the paper is the variance cost function, as shown in the following equation:

(14) $$\begin{array}{c} J\left(W,b\right)=\left[\frac{1}{m}\sum_{i=1}^{m}\frac{1}{2}{\left\|h_{w,b}\left(x^{i}\right)-y^{i}\right\|}^{2}\right]+\frac{\lambda }{2}\sum_{l=1}^{L}\sum_{i=1}^{i}\sum_{j=1}^{j}{\left(W_{ij}^{l}\right)}^{2}. \end{array}$$

*λ* is a weight adjustment factor to adjust the balance of the two terms before and after the *J*(*W*, *b*) plus sign. When the latter term is more important, the size of *λ* increases. The residuals of this error function are calculated as shown in the following equation:

(15) $$\begin{array}{c} \delta_{i}^{n}=\frac{\partial }{\partial Z_{i}^{n}}\frac{1}{2}{\left\|y-h_{w,b}\left(x\right)\right\|}^{2}=-\left(y_{i}-a_{i}^{n}\right)\cdot f^{\prime }\left(Z_{i}^{n}\right). \end{array}$$

### 2.4. Medical Diagnosis Model Construction Based on Integrated Deep Neural Network Algorithm

**Figure 5:**
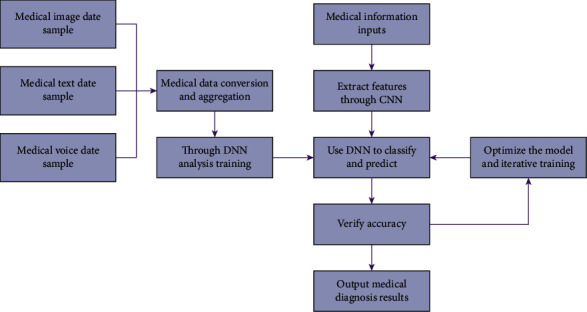
Intelligent medical diagnosis model.

Common medical diagnosis includes the processing of images, natural language, and speech, and the medical diagnosis model is built based on the integrated deep neural network algorithm, as shown in Figure 5.

We give examples of the results of processing computer images and natural text in a deep neural system, respectively.

**Figure 6:**
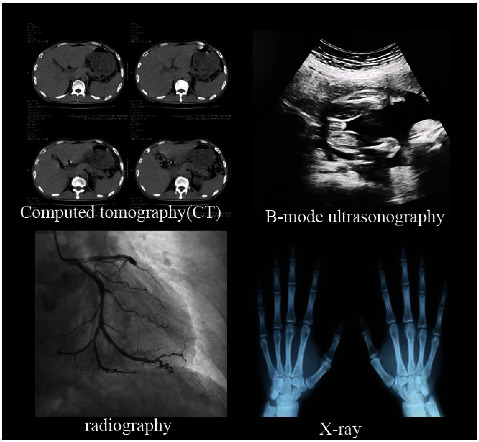
Legend of common medical images.

In the daily medical examination, we often carry out various types of impact images, so in the process of training the intelligent diagnosis system, it is necessary to input various types and parts of the medical in images for training and optimization, and common medical images are shown in Figure 6.

**Figure 7:**
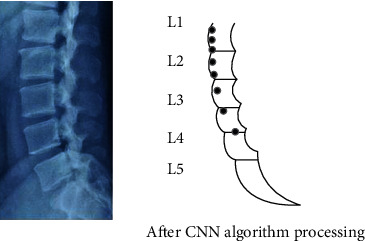
CT image processed by the CNN algorithm.

The CT diagnostic image is taken as an example, as in Figure 7; we selected the CT image of the lumbar spine and compared it before and after processing by the CNN algorithm. After the algorithm processing, we can more obviously find the lumbar spine curvature of the patient and determine the cause of the disease.

**Figure 8:**
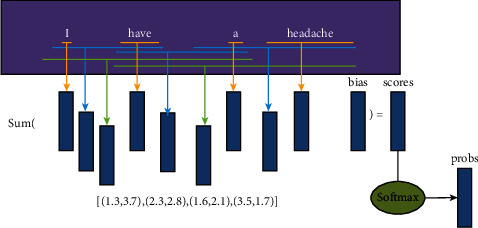
Transformation of the natural language in the algorithm.

The natural language input from a human is taken as an example, as shown in Figure 8. This two-dimensional matrix can be used in the subsequent model training.

In the following section, we will simulate and evaluate this model using natural language as an example.

#### 2.4.1. Data Preprocessing

Before the deep neural network model training, we need to preprocess the data, which are mainly from the following sources: (1) hospital database; (2) web-based medical data; and (3) authoritative medical literature.

We use real patient cases as model training data and then add comprehensive medical records from the authoritative literature to the training data through regularization and rule collation to supplement the above condition data. Here, we first use the Chinese word splitting tool Jieba to split the symptoms presented in the patient cases to separate the nouns accurately and extract them.

In order to compensate for the inability of this word-sorting tool to accurately cut the symptoms and to judge the attributes of the symptoms themselves, we constructed and supplemented the lexicon, such as adding the description of “negative” symptoms. After the improvement, we filtered and deleted the symptom descriptions by word separation and kept the samples with the number of symptoms greater than or equal to 2 according to the actual situation so as to make the diagnosis more rigorous. Finally, we retained 13,571. Since there are multiple disease diagnostic names in the diagnostic word set of each data sample, when making disease diagnosis, we need to pay attention to the vocabulary name of the disease to avoid wrong diagnosis.

However, after finishing the medical data, we need to convert the data into a form that can be recognized and processed by deep neural networks, so in the second step, the diagnostic data need to be transformed, and in this paper.

The last step of data preprocessing is to aggregate the data, and the retained 13,571 samples are transformed into 724-dimensional word frequency statistics data type and disease diagnosis into 1006-dimensional one-hot data type.

#### 2.4.2. Model Predictions

In this model construction, we build two neural network models, which are fully connected neural networks and convolutional neural networks, and traditional decision tree models, such as Dropouts meet Multiple Additive Regression Trees. For the convolutional neural network model, the input data can be well fed back to the network. For the convolutional neural network model, the input data are fed into the network, which can automatically perform feature extraction and then connect it to the fully connected neural network for classification prediction.

In the fully connected neural network model, the input layer that is 724 neurons, against which there are 724 disease symptoms, and the output layer that is 1006 neurons correspond to 1006 diseases, respectively. In the hidden layer of this model, we apply the ReLU activation function, which updates the parameters with the best effect and helps us to effectively prevent the problem of gradient disappearance in the process of gradient descent to update the parameters.

In the output layer of the model, we use the softmax activation function to classify diseases and thus predict the probability of occurrence of various diseases.

For the evaluation function, we chose top_*k*_accuracy, which is considered correct when the true disease outcome is in the top *K* (*k* = 10 for this experiment) positions of the predicted disease outcome probability.

The Dropout layer is used to randomly drop out the neurons to prevent overfitting. The Dropout value of 0.5 is chosen for this model. Finally, iterations are performed in the neural network model, and in general the more the iterations are, the higher the corresponding accuracy will be, but excessive iteration can also cause the overfitting phenomenon, so we control the number of the iterations epoch number (epoch = 15 is chosen for this experiment).

#### 2.4.3. Model Evaluation

**Figure 9:**
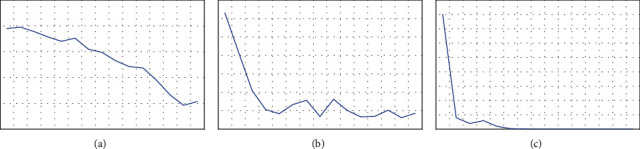
Cross-entropy loss curve change: (a) Dropouts meet Multiple Additive Regression Trees loss, (b) DNN loss, and (c) CNN loss.

The above three types of neural network models were evaluated to compare the stability, accuracy, and error cause analysis of the three models.  Figure 9 shows the trend of the cross-entropy loss values of the prediction results of the three functional models after training to reflect the error magnitude.

From Figure 9, we can see that the trend of cross-entropy loss value of different models is different, we should choose the model with small and smooth cross-entropy loss value for training, through observation and comparison, we found that the traditional system tree is the slowest decline, DNN although the decline is fast but the network fluctuates a lot, very unstable, CNN's is not only the fastest decline, and close to 0, the network state It is very stable.

**Figure 10:**
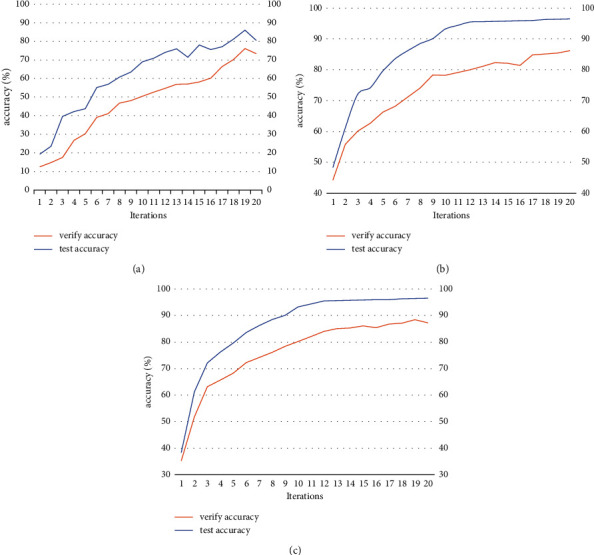
Model accuracy convergence diagram: (a) Dropouts meet Multiple Additive Regression Trees accuracy, (b) DNN accuracy, and (c) CNN accuracy.

Figure 10 shows the accuracies of each of the three models. It is not difficult to find that Dropouts meet Multiple Additive Regression trees has the lowest accuracy, and among the deep neural network models, the model with the addition of the coiled cypress neural network is more accurate and more realistic than the fully connected deep neural network.

The deep neural network models in this experiment were all found to be overfitted to some extent during the evaluation process, and the reasons for this are analyzed as follows.

**Figure 11:**
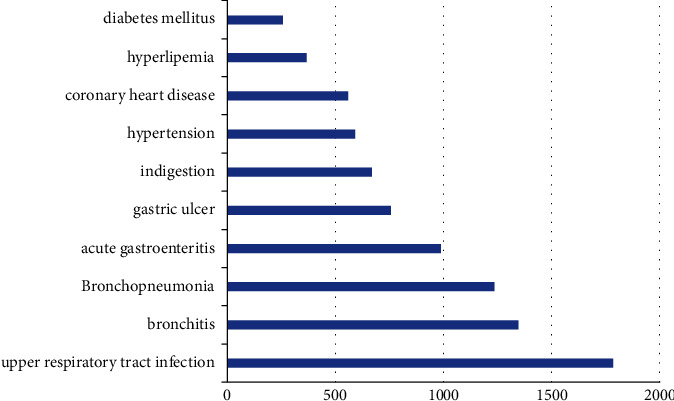
Number of high frequency diseases.

**Figure 12:**
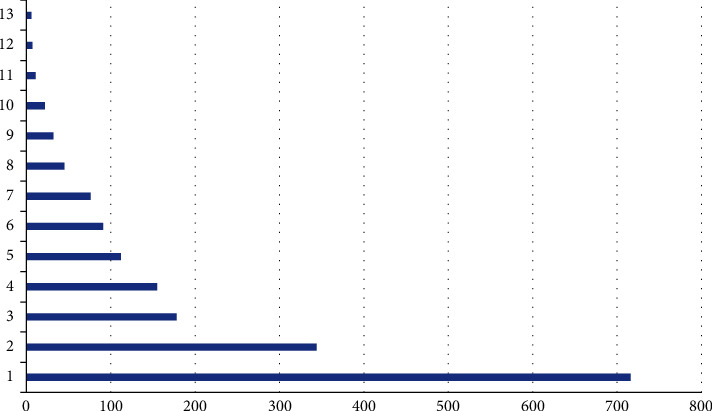
Frequency of low frequency diseases.

This paper studies the distribution of some medical data; due to the effective number of collections, the number of data in the sample is not evenly distributed, and the number of many rare diseases or diseases whose causes have not yet been found is too small, resulting in the machine not being able to learn sufficiently and eventually inaccurate disease diagnosis, with specific disease distribution as shown in Figures 11 and 12.

In this paper, the neural network model contains many kinds of diseases and it is difficult to adjust the parameters, so the model design is too complex, but the sample data are relatively too small, which leads to the overfitting phenomenon.

#### 2.4.4. Analysis of Model Results

After the above model evaluation, we found that compared with the traditional decision tree, the deep neural network model has higher stability and accuracy, with an accuracy rate of 96%, and is more suitable for medical diagnosis, especially the rolled cypress neural network system, which has stronger advantages in language and text images, can reduce the parameters of the network and improve the training efficiency of the neural network. However, this experiment is too complex due to the model, the feature dimension is too large, and the amount of data is not sufficient, which to some extent leads to the main feature extraction effect of the volume cypress neural network on the data which is not optimal, and further improvement is needed in the subsequent experiments.

## 3. Conclusion

This study proposes a set of intelligent medical diagnosis models based on integrated deep neural network algorithms. By combining medical big data, the authoritative expert literature, and neural network algorithms, it makes up for many problems in the traditional medical system, gives full play to its advantages, and improves the stability and accuracy of medical diagnosis. Through experimental verification, it is found that the intelligent medical diagnosis model built in this paper based on the rolled-up neural network has higher accuracy and efficiency when compared with the traditional decision tree.

For the diagnosis of diseases, it can reach or even exceed the diagnosis results of real doctors and even be more efficient and convenient, saving a lot of trouble on medical treatment. However, to build an intelligent medical big data platform that can be shared by the whole society, the model design must ensure sufficient disease types and sample data volume so that the machine can fully learn and reduce the error degree. The intelligent medical diagnosis model based on integrated deep neural network built in this paper can systematically evaluate and analyze the symptoms that patients present and provide a theoretical basis for big data algorithms to prevent other diseases and further improve and explore the intelligent medical neighborhood. In summary, this experiment gives us a glimpse of the future hope to see a deeper use of integrated deep neural network algorithms and medical neighborhoods combined to promote smart Healthcare imaging and infrastructure to help more people enjoy quality Healthcare imaging services.

## Data Availability

The data used to support the findings of this study are available from the corresponding author upon request.
